# Supplementary material for: Klf4 glutamylation is required for cell reprogramming and early embryonic development in mice
Source: Nat Commun. 2018 Mar 28;9:1261. doi: 10.1038/s41467-018-03008-2 (PMC5871780; doi:10.1038/s41467-018-03008-2)
Supplement: Supplementary file 1 — Supplementary Information [file 41467_2018_3008_MOESM1_ESM.docx]

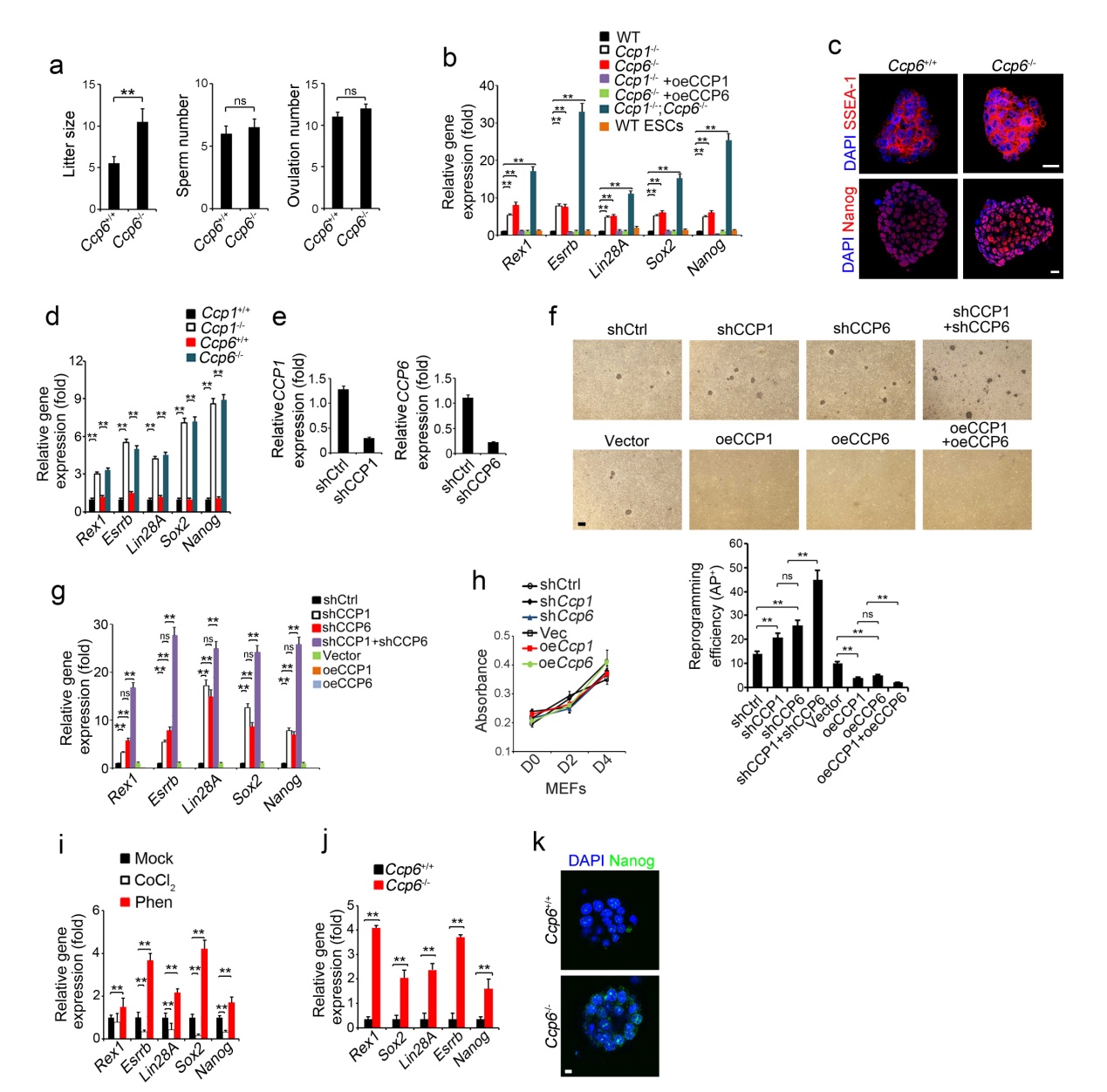


**Supplementary Figure 1. CCP1 or CCP6 deficiency upregulates pluripotent gene expression in the process of reprogramming.** (a) Mice were naturally mated. Litter size at birth were counted as means ± S.D. Sperm was isolated from cauda epididymes and counted as means ± S.D. Superovulation eggs were collected by flushing oviducts and counted as means ± S.D. **, *P*<0.01. ns, no significance. n=10. (b) WT or CCPs deficient MEFs were induced iPS formation as in 1 (b). 5×10^5^ iPS cells were sorted and mRNAs levels of indicated genes were analyzed by real time qPCR. WT ESCs were used as a positive control. Relative gene expression fold changes were counted as means ± S.D. **, *P*<0.01. n=5. Primer pairs are shown in Supplementary Table 1. oe, overexpression. (c) MEFs were induced with OSKM factors for 3 weeks and immunostained with anti-SSEA-1 or anti-Nanog antibodies. Nuclei were stained by DAPI. Scale bar, 20 μm. (d) iPSCs were isolated and expanded in the absence of feeders. mRNAs levels of the indicated genes were analyzed as in (b). Relative gene expression fold changes were counted as means ± S.D. **, *P*<0.01. n=5. (e) CCP1 or CCP6 depletion in MEFs was confirmed by real-time qPCR. (f) MEFs were infected by OSKM factors containing retrovirus and the indicated shRNA as in (b). Alkaline phosphatase (AP) positive colony numbers per 10^4^ cells were calculated and shown as means ± S.D. **, *P*<0.01. n=5. Scale bar, 100 μm. (g) MEFs were induced iPSC formation as in (b) followed with real time qPCR. Relative gene expression fold changes were counted as means ± S.D. **, *P*<0.01. n=5. (h) MEFs were seeded 1×10^3^ per well, CCK-8 was added to culture medium every day and cell proliferation were counted. (i) MEFs were treated with CoCl_2_ (10 μM) or phenanthroline (Phen, 1μM) in ESC media for iPSC formation as in (b), followed by gene expression assays. Relative gene expression fold changes were counted as means ± S.D. **, *P*<0.01. n=5. (j) Embryos were isolated at E1.5 stage and cultured as in 1 (f). mRNAs were analyzed as in (b). Relative gene expression fold changes were counted as means ± S.D. **, *P*<0.01. n=5. (k) Embryos were isolated and immunostained with anti-Nanog antibody. Nuclei were stained by DAPI. Scale bar, 20 μm. Student’s *t*-test was used as statistical analysis. Data are representative of three independent experiments.


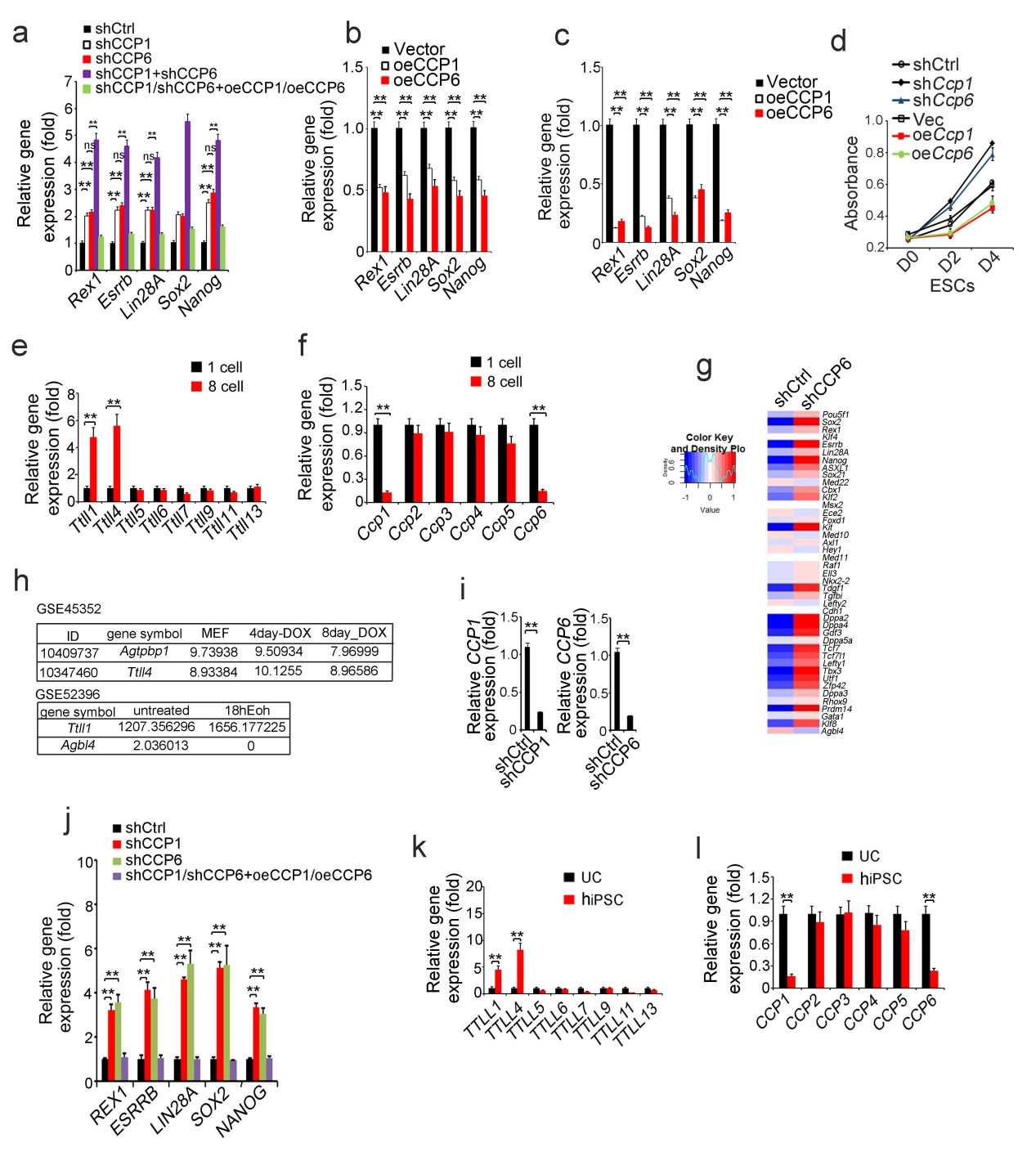


**Supplementary Figure 2. CCP1 and CCP6 regulate the maintenance of murine and human pluripotency.** (a) Mouse ES cell line D3 cells were transfected with the indicated plasmids and cultured in mouse ESC media for 3 days. mRNAs levels of the indicated genes were analyzed by real time qPCR. Relative gene expression fold changes were counted as means ± S.D. **, *P*<0.01. ns, no significance. n=5. Primer pairs are shown in Supplementary Table 1. (b) D3 cells were transfected with the indicated plasmids. mRNAs levels of the indicated genes were analyzed as described in (a). Data represent four independent experiments are shown as means ± S.D. **, P<0.01. n=5. (c) R1 ES cells were transfected with the indicated plasmids. mRNAs levels of the indicated genes were analyzed as described in (a) and shown as means ± S.D. **, P<0.01. n=5. (d) CCP1/CCP6 depletion promoted proliferation of ESCs. Proliferation was measured by CCK-8 staining as in 1(h). (e, f) mRNAs of TTLLs and CCPs were analyzed in 1 cell and 8 cell stage embryos by real time qPCR. Relative gene expression folds were normalized to endogenous β-actin and shown as means ± S.D. **, *P*<0.01. n=5. (g) Mouse ES cell line D3 cells were transfected with the shCCP6 plasmids and cultured in mouse ESC media for 3 days. mRNAs levels of indicated genes were analyzed by gene expression profile chip assay (Affimatrix, GSE106809). (h) Expression changes of *Ccp1* (*agtpbp1*), *Ttll4*, *Ttll1* and *Ccp6* (*Agbl4*) during reprogramming were analyzed from RNAseq datasets GSE45352 and GSE52396. (i) CCP1 or CCP6 depletion in human iPSCs was confirmed by real-time qPCR. Relative gene expression fold changes were counted as means ± S.D. **, *P*<0.01. (j) Human iPSCs H9 or H1 were infected with indicated lentivirus cultured for 3 weeks followed by real time qPCR. Relative gene expression fold changes were counted as means ± S.D. **, *P*<0.01. n=5. (k, l) mRNAs of TTLLs and CCPs were analyzed in human urothelial cells (UC) and human iPSCs by qPCR. Relative gene expression fold changes were counted as means ± S.D. **, *P*<0.01. n=5. Student’s *t*-test was used as statistical analysis.


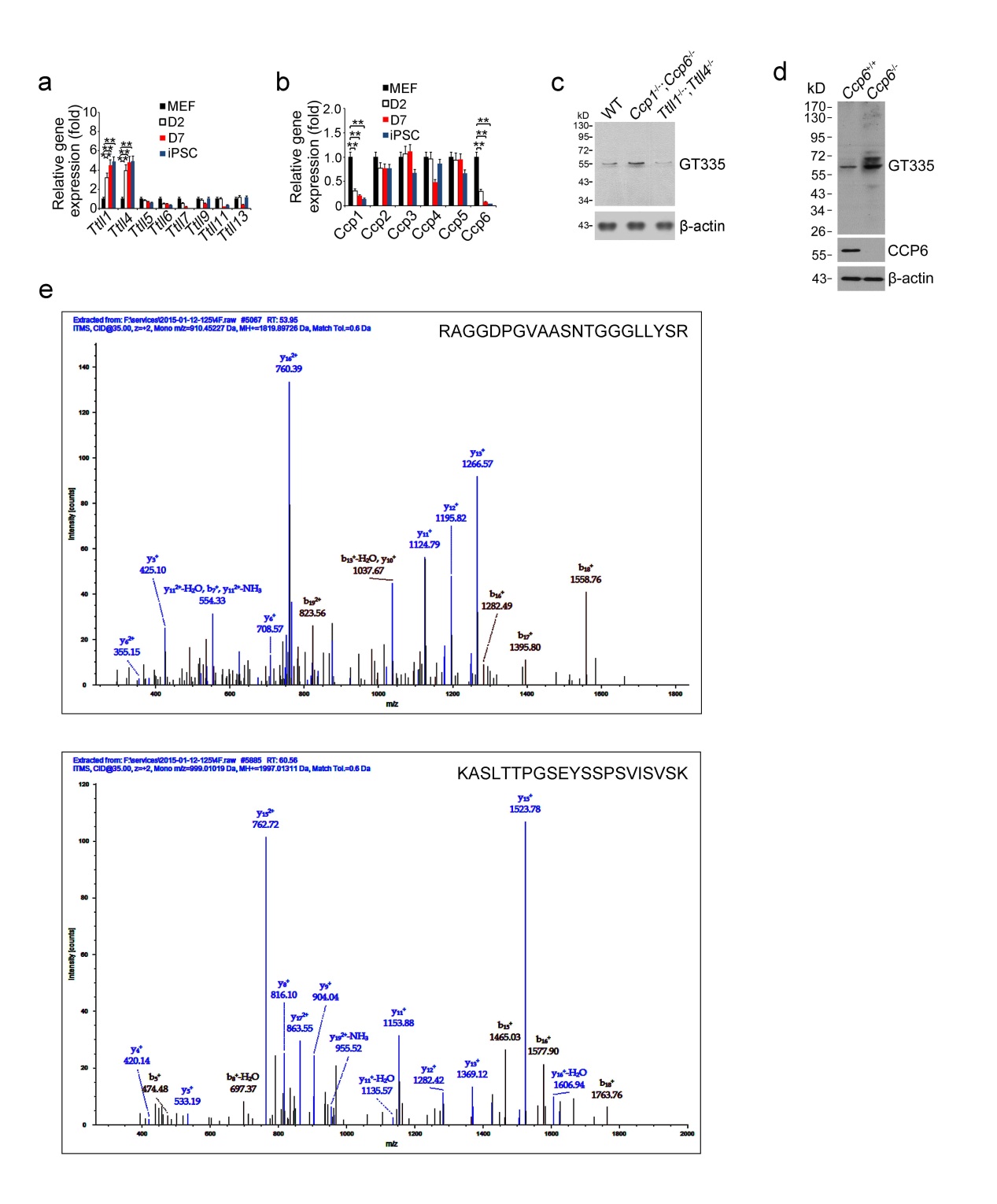


**Supplementary Figure 3. Klf4 undergoes glutamylation in iPSC generation.** (a, b) mRNAs levels of TTLLs (a) and CCPs (b) during iPSC induction were analyzed by real time qPCR. Relative gene expression folds were normalized to endogenous β-actin and shown as means ± S.D. **, *P*<0.01. n=5. Primer pairs are shown in Supplemental Supplementary Table 1. (c) The specificity of GT335 antibody was verified by immunoblotting. WT, CCPs or TTLLs deficient MEFs were lyzed and analyzed by immunoblotting with GT335 antibody. (d) MEF lysates were probed with the indicated antibodies. (e) Identification of Klf4 as a glutamylation substrate. Klf4 protein sequences were identified by LTQ Orbitrap XL. Student’s *t*-test was used as statistical analysis. Data are representative of four independent experiments.


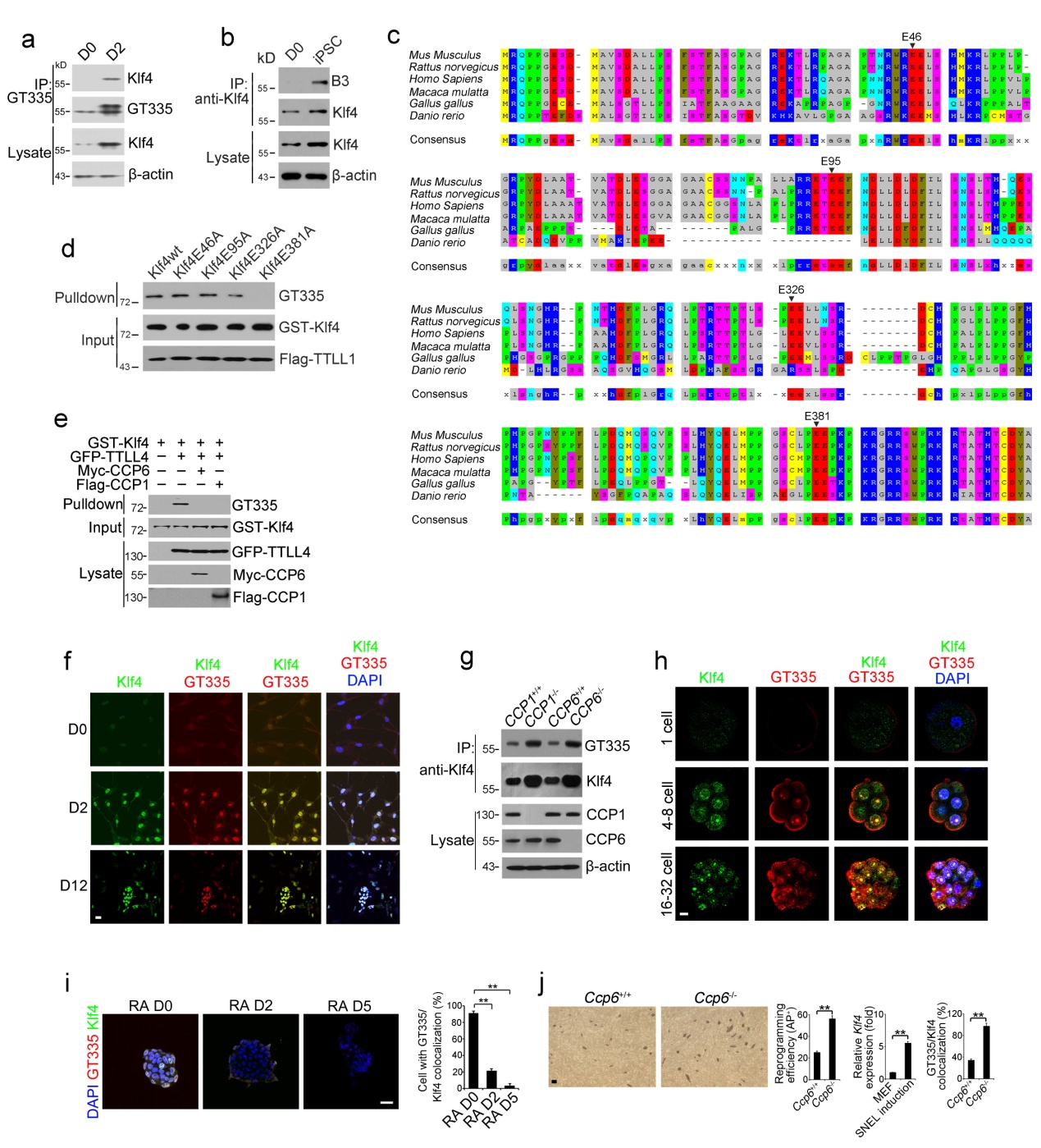


**Supplementary Figure 4. Glu381 is the acceptor site for glutamylation of Klf4.** (a) MEF lysates induced by OSKM for the indicated days were immunoprecipitated with GT335 antibody and analyzed by immunoblotting with anti-Klf4 antibody. (b) Cells were lysed by RIPA lysis buffer, and incubated with anti-Klf4 antibody with protein A/G beads, followed by immunoblotting with B3 antibody. (c) Sequence analysis of Klf4 Glu-rich region. (d) Flag-tagged TTLL1 together with OSKM factors was expressed in MEFs. GST-Klf4wt and indicated mutant proteins were incubated with Flag-TTLL1. GST-Klf4 proteins were pulled down by Glutathione Sepharose 4B beads, followed by immunoblotting. (e) MEFs were infected with GFP-TTLL4, Myc-CCP6 and Flag-CCP1 retrovirus. Lysates were incubated with GST-Klf4 for 2 h for *in vitro* deglutamylation followed by GST pulldown assay. (f) MEFs were induced with OSKM factors and immunostained with anti-Klf4 and GT335 antibodies. Nuclei were stained by DAPI. Scale bar, 20 μm. (g) Indicated MEFs were transduced with OSKM and immunoprecipitated with anti-Klf4 antibody, followed by immunoblotting. (h) Embryos for the indicated days were isolated and immunostained with anti-Klf4 and GT335 antibodies. Nuclei were stained by DAPI. Scale bar, 20 μm. (i) Mouse D3 ES cells were induced differentiation by retinoid acid (RA, 1 μM) treatment for the indicated days and immunostained with anti-Klf4 and GT335 antibodies. Nuclei were stained by DAPI. Scale bar, 20 μm. The colocalization between Klf4 and GT335 signals were calculated and shown as means ± S.D. **, *P*<0.01. 112 typical ES clones for D0, 105 typical ES clones for D2, 120 typical ES clones for D5 were observed. (j) WT or CCPs deficient MEFs were infected by SNEL (Sall4, Nanog, Esrrb and Lin28) factors containing retrovirus and cultured in ESC media for 3 weeks. Alkaline phosphatase (AP) positive colony numbers per 10^4^ cells were calculated and shown as means ± S.D. **, *P*<0.01. Klf4 mRNA expression was assessed 2 days after SNEL induction. Relative gene expression fold changes were counted as means ± S.D. **, *P*<0.01. Data represent four independent experiments (n=4). Cells were immunostained with anti-Klf4 and GT335 antibody. The colocalization between Klf4 and GT335 signals were calculated and shown as means ± S.D. **, *P*<0.01. 150 typical cells were observed. Student’s *t*-test was used as statistical analysis.


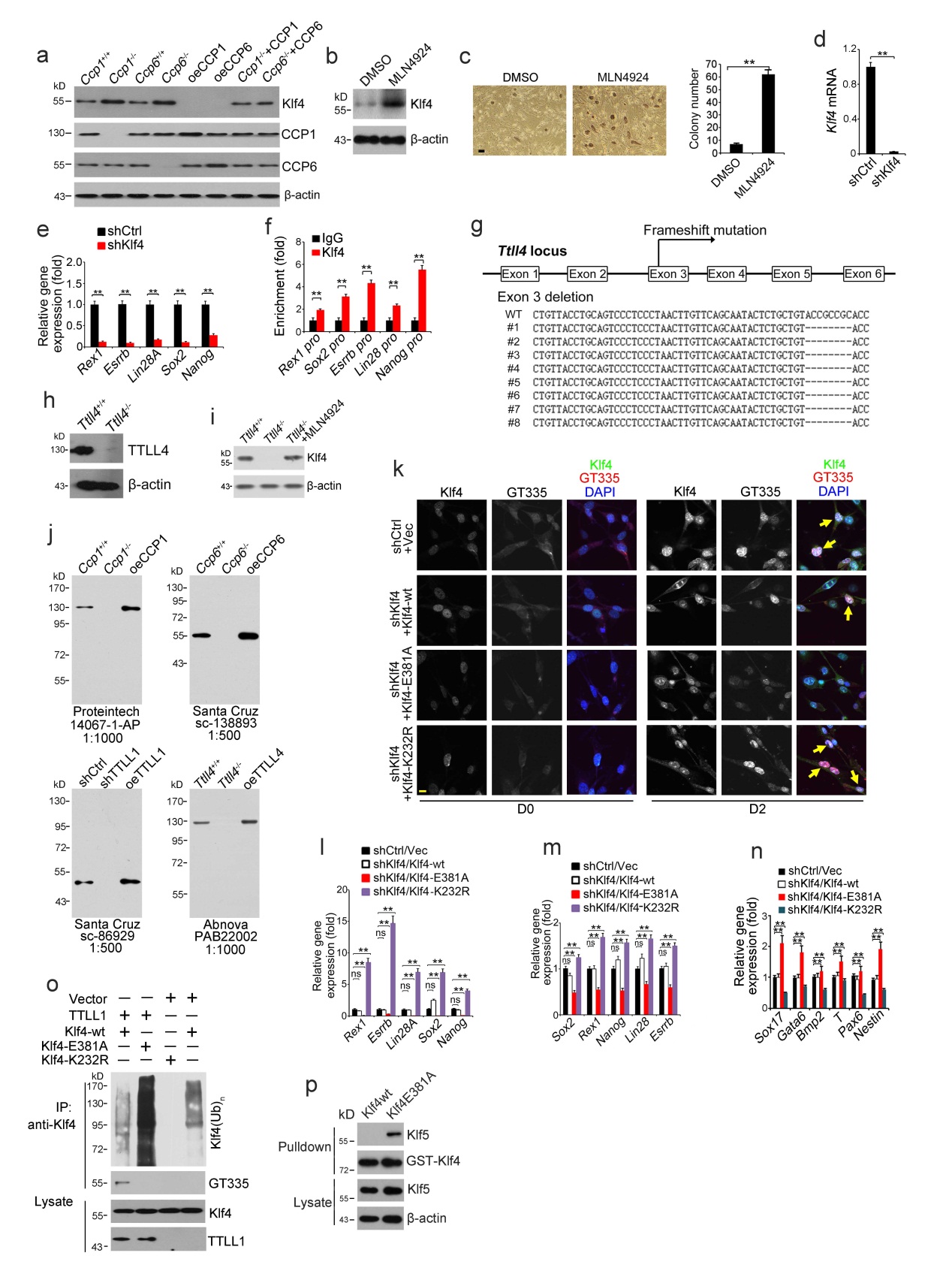


**Supplementary Figure 5. Overexpresson of CCP1 or CCP6 promotes Klf4 protein degradation over cell reprogramming.** (a) MEFs were infected by OSKM factors in indicated lentivirus. Klf4 was probed by immunoblotting. (b) WT MEFs were transfected by OSKM factors plus MLN4924 (1 μM ) treatment for 5 days. Cells were lyzed for immunoblotting. (c) MEFs were induced with OSKM lentivirus and treated with 1 μM MLN4924 for 3 weeks. AP positive colony numbers per 10^4^ cells were calculated and shown as means ± S.D. **, *P*<0.01. n=5. Scale bar, 100 μm. (d) Klf4 depletion was confirmed by qPCR. Relative gene expression was shown as means ± S.D. **, P<0.01.n=3. (e) Mouse ES D3 cells were transfected with the indicated plasmids followed by qPCR. Relative gene expression was shown as means ± S.D. **, P<0.01. n=5. (f) Accumulation of Klf4 on promoter of pluripotency gene was confirmed by ChIP-qPCR. Fold enrichment was shown as means ± S.D. **, *P*<0.01. n=5. (g) Diagram of the strategy for *Ttll4* targeting via CRISPR-Cas9 technology. (h) TTLL4 deficiency was confirmed by immunoblotting in MEFs. (i) MEFs were infected by OSKM factors with MLN4924 (1 μM) treatment for 5 days and lyzed for immunoblotting. (j) The specificities of antibodies were verified by immunoblotting. (k) Klf4-wt, Klf4-E381A or Klf4-K232R were overexpressed into Klf4 silenced MEFs after OSKM transfection for 2 days, followed by staining with anti-Klf4 antibody and GT335. Klf4, green; GT335, red; DAPI, blue. Scale bar, 10 μm. More than 100 typical cells were assessed. Yellow arrow indicates cell with Klf4/GT335 colocalization. (l) mRNAs levels of the indicated genes in above treated MEFs as in (k) were analyzed by real time qPCR. Relative gene expression changes were calculated as means ± S.D. **, *P*<0.01. ns, no significance. n=4. (m-n) mRNAs levels of the indicated genes in D3 cells were analyzed by real time qPCR. Relative gene expression changes were calculated as means ± S.D. **, *P*<0.01. ns, no significance. n=5. (o) MEFs were infected with OSKM factors and indicated lentivirus followed by immunoprecipitation. (p) Klf4-E381A mutant is dimerized with Klf5 in ESCs. ESCs were lyzed and incubated with indicated protein, followed by GST pulldown assay. Student’s *t*-test was used as statistical analysis. Data are representative of four independent experiments.


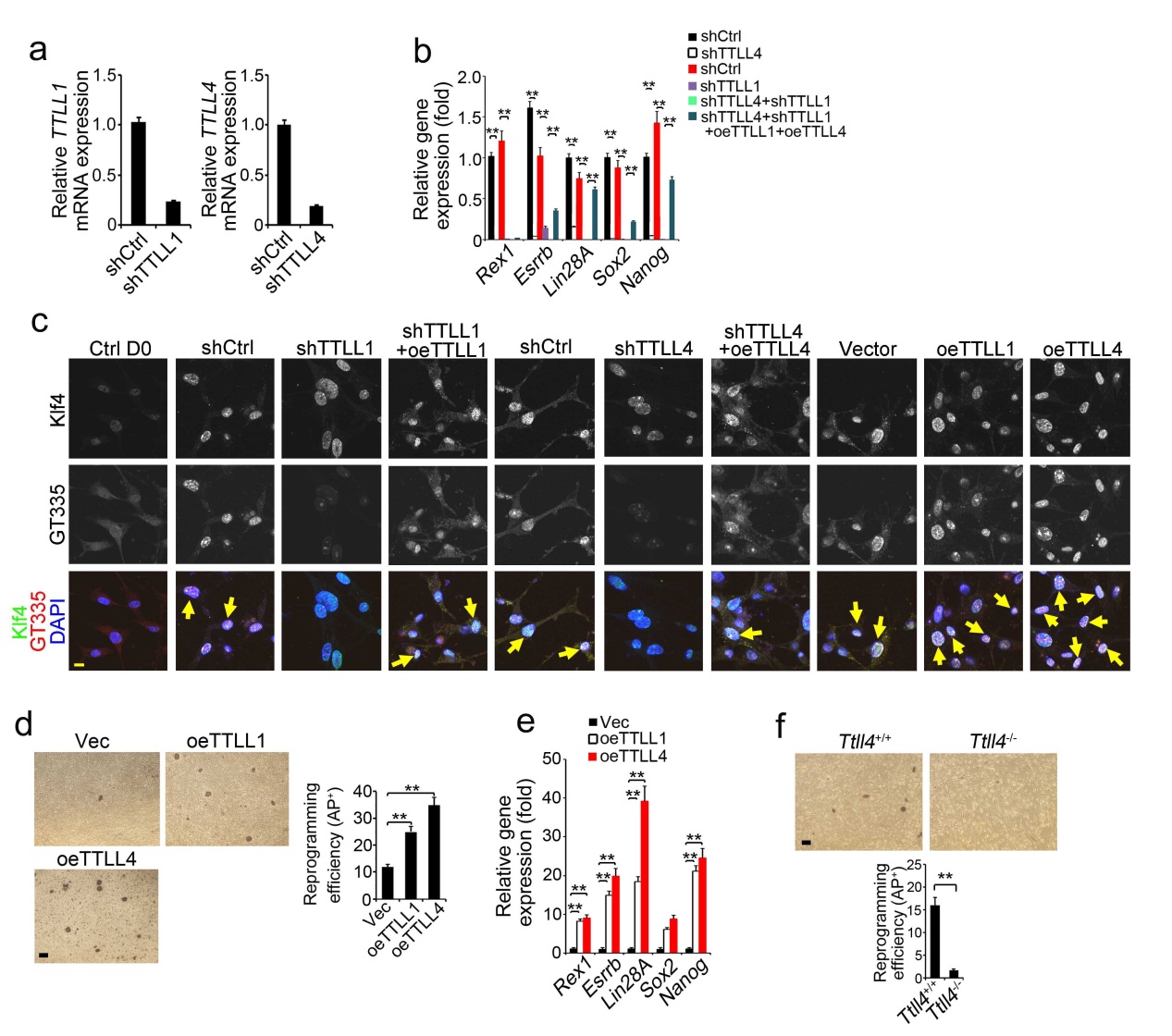


**Supplementary Figure 6. Depletion of TTLL1 and TTLL4 impairs cell reprogramming.** (a) TTLL1 or TTLL4 depletion in MEFs was confirmed by real-time qPCR. (b) TTLL1 or TTLL4 depletion downregulates pluripotent gene expression during mouse iPSC formation. MEFs were transfected by OSKM factors with the indicated plasmids and induced iPSC formation as described in Supplementary Figure 1b. mRNAs levels of the indicated genes were analyzed by real time qPCR. Relative gene expression fold changes were counted as means ± S.D. **, *P*<0.01. n=4. Primer pairs are shown in Supplementary Table 1. oe, overexpression. (c) Assessment of Klf4 and GT335 colocalization in above treated MEFs (b) by staining with anti-Klf4 antibody (green), GT335 (red), and DAPI (blue). Scale bar, 10 μm. Yellow arrow denotes the cell with Klf4/GT335 colocalization. More than 100 typical cells were assessed. (d) Overexpression of TTLL1 or TTLL4 promotes iPSC formation. MEFs were transfected by OSKM factors plus the indicated plasmids and induced iPSC formation as described in Supplementary Figure 1b. AP positive colony numbers per 10^4^ cells were calculated and shown as means ± S.D. **, *P*<0.01. n=5. Scale bar, 100 μm. (e) mRNAs levels of the indicated genes in above treated MEFs were analyzed by real time qPCR as described in (b). Relative gene expression fold changes were counted as means ± S.D. **, *P*<0.01. n=5. (f) TTLL4 deficiency impairs iPSC formation. *Ttll4*^+/+^ or *Ttll4*^-/-^ MEFs were infected by OSKM factors containing retrovirus and induced iPSC formation as described in Fig S1b. AP positive colony numbers per 10^4^ cells were calculated and shown as means ± S.D. **, *P*<0.01. n=5. Scale bar, 100 μm. Student’s *t*-test was used as statistical analysis.


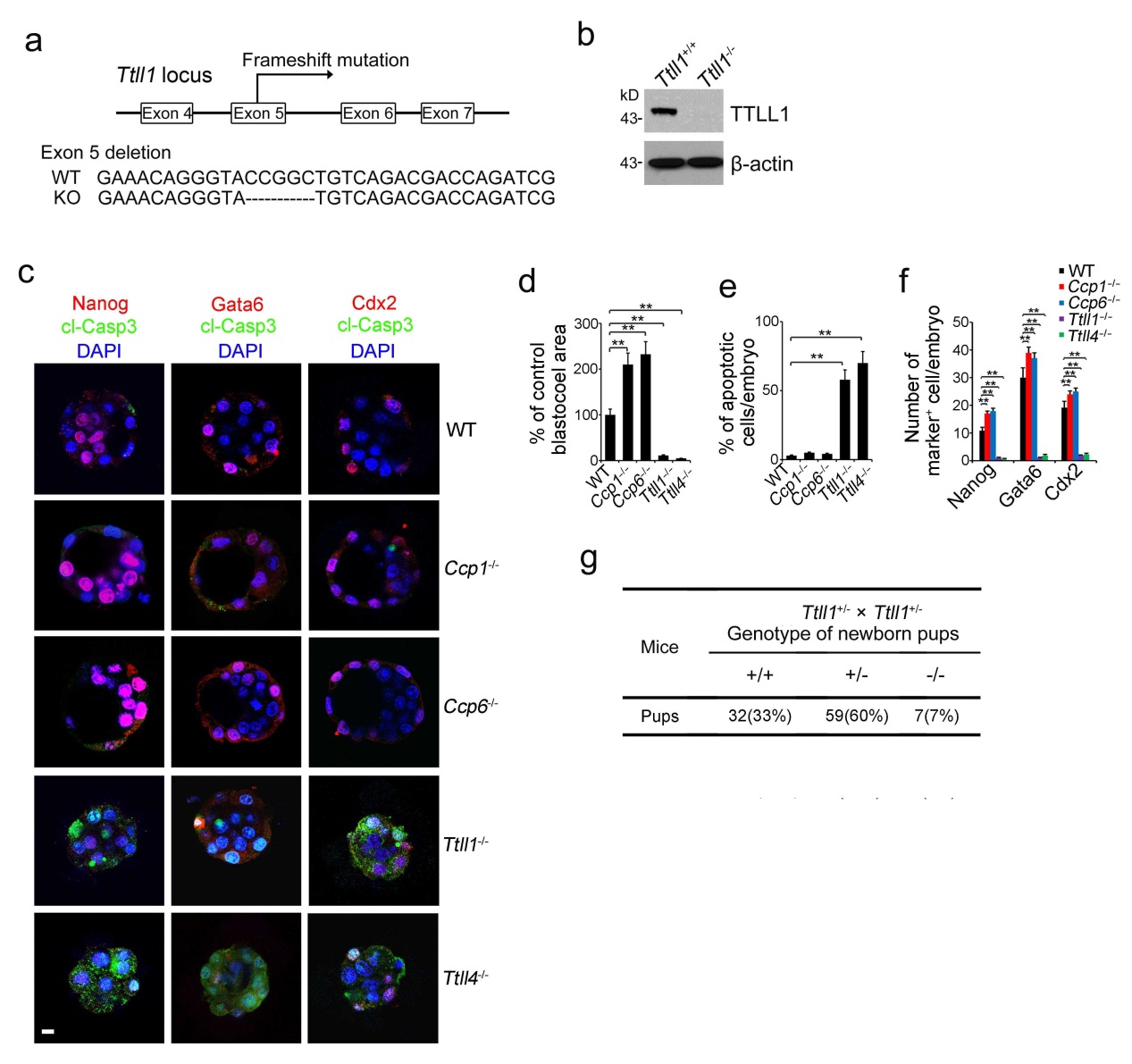


**Supplementary Figure 7. Depletion of TTLL1 and TTLL4 impairs embryonic development.** (a) Diagram of the strategy for *Ttll1* targeting via CRISPR-Cas9 technology. A 5 bp deletion of exon 5 (frameshift mutation) was identified by PCR screening and DNA sequencing. (b) TTLL1 deficiency was confirmed by immunoblotting in MEFs. (c) Indicated preimplantation embryos were stained with cell fate markers (Nanog for epiblast (EPI), Gata6 for primitive endoderm (PrE), Cdx2 for trophectoderm (TE)) as well as apoptosis marker cleaved caspase 3 (cl-Casp3). Scale bar, 10 μm. (d) Blastocoel area variety compared with wt mice was calculated as means ± S.D. **, *P*<0.01. For WT embryos, n=105. For *Ccp1*^-/-^ embryos, n=102. For *Ccp6*^-/-^ embryos, n=127. For *Ttll1*^-/-^ embryos, n=104. For *Ttll4*^-/-^ embryos, n=107. (e) Number of active caspase 3 positive cell per embryo was calculated as means ± S.D. **, *P*<0.01. For WT embryos, n=75. For *Ccp1*^-/-^ embryos, n=81. For *Ccp6*^-/-^ embryos, n=57. For *Ttll1*^-/-^ embryos, n=44. For *Ttll4*^-/-^ embryos, n=51. (f). Number of lineage marker positive cell per embryo was calculated as means ± S.D. **, *P*<0.01. For WT embryos, n=35. For *Ccp1*^-/-^ embryos, n=31. For *Ccp6*^-/-^ embryos, n=37. For *Ttll1*^-/-^ embryos, n=34. For *Ttll4*^-/-^ embryos, n=28(anti-Nanog staining). For WT embryos, n=44. For *Ccp1*^-/-^ embryos, n=21. For *Ccp6*^-/-^ embryos, n=32. For *Ttll1*^-/-^ embryos, n=24. For *Ttll4*^-/-^ embryos, n=28(anti-Gata6 staining). For WT embryos, n=45. For *Ccp1*^-/-^ embryos, n=38. For *Ccp6*^-/-^ embryos, n=35. For *Ttll1*^-/-^ embryos, n=24. For *Ttll4*^-/-^ embryos, n=27(anti-Cdx2 staining). (g) *Ttll1*-deficient pups were genotyped after heterozygotes crossing. Student’s *t*-test was used as statistical analysis. Data are representative of four independent experiments.


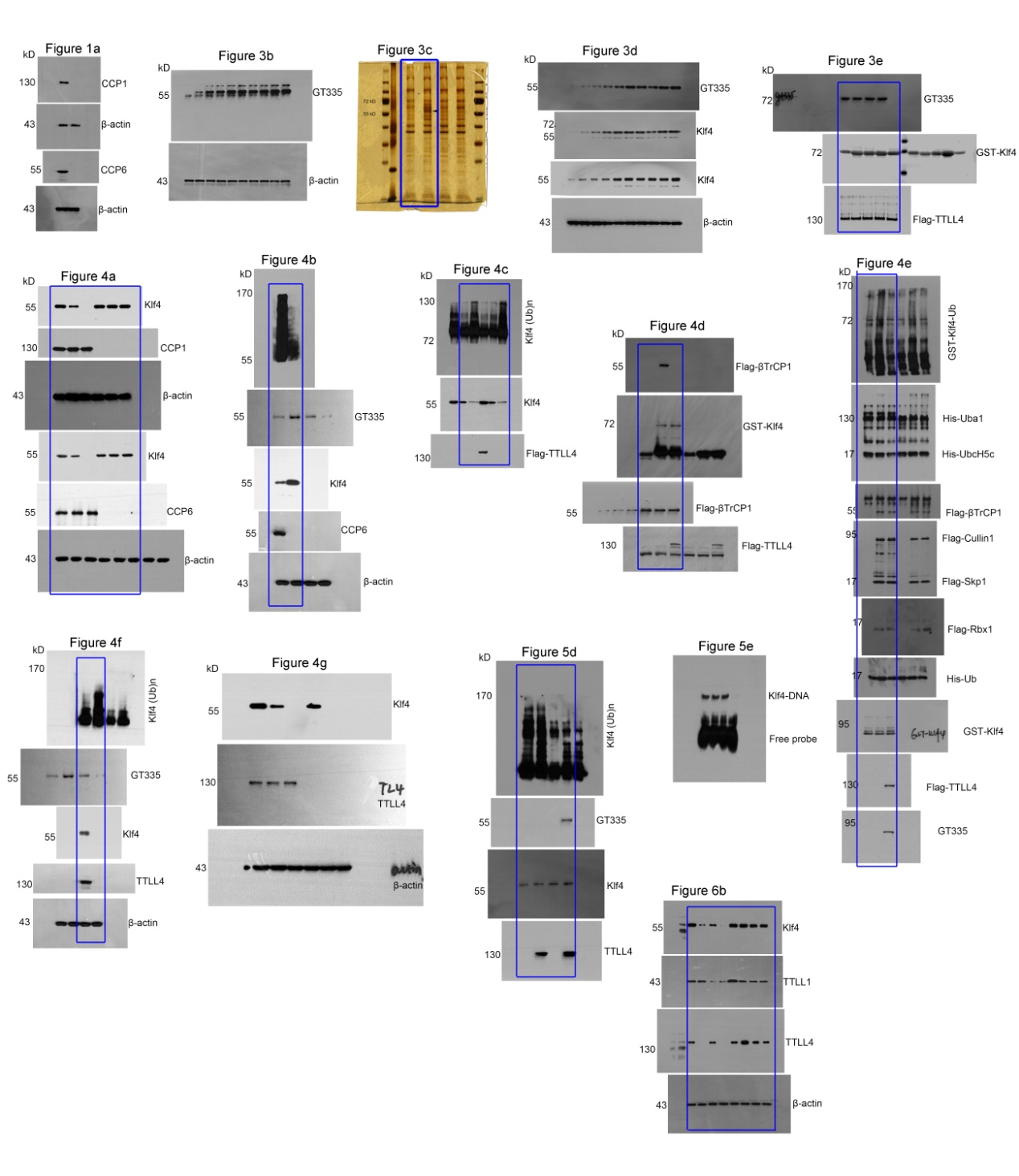


**Supplementary Figure 8. Uncropped images of western blotting and gel in figure 1-7.**

**Supplementary Table 1. Primer oligonucleotides for qPCR**

| Genes | Forward | Reverse |
| --- | --- | --- |
| mCCP1 | 5’- GGGGTCGAAGAGCGAGTTTC-3’ | 5’- GAATGGAGTGAGTCTGCACCA-3’ |
| mCCP2 | 5’-ATGAATGTCCTGCTTGAGATGG-3’ | 5’-CAAACGCGCTGATGAGTGC-3’ |
| mCCP3 | 5’-AGCTGAAGATGCTTACAAAGAGC-3’ | 5’-CGCACAGTCAACTCGTATTCAT-3’ |
| mCCP4 | 5’-CCAGCAGTGCCTATACCTTCC-3’ | 5’-TGCTCAGATCAGTTTCCAAGTC-3’ |
| mCCP5 | 5’-CTGCTCATTCTCGTCTTCAGG-3’ | 5’-ATCGAGTCCTAATGCAAGGGA-3’ |
| mCCP6 | 5’- AGGCAGGCAATGATACAGGAA-3’ | 5’- GGTTACCACTTTCAAAGCAAGCA-3’ |
| mTTLL1 | 5’- GAAGTGGGTCACTGACATTGAG-3’ | 5’- ACGTTGCGAATGGTTTGCAC-3’ |
| mTTLL4 | 5’- TGGATGAGAACCTGAAACCCT-3’ | 5’- TGGGGCTGCTGGAACTAGA-3’ |
| mTTLL5 | 5’- ACTCCCCAGCTCCCATCTG-3’ | 5’- GGGGCATTGTCAGGAACGG-3’ |
| mTTLL6 | 5’- AAGCCCTTCATCATCGACGG-3’ | 5’- TGTCTAGGTTAGGGTGGGAGTAA-3’ |
| mTTLL7 | 5’- CTCTGCCTCAAGATGGGGTTA-3’ | 5’- GTTCCGGCAACATTAGCTGTAA-3’ |
| mTTLL9 | 5’- TGGAGTGTCGAAAGGAAAAGAGA-3’ | 5’- TGCTCATCCATGTAGGTGTGG-3’ |
| mTTLL11 | 5’- CCTGACCAACTACTCCCTGAA-3’ | 5’- GGGATGTCTGACTGGTAGAAAAC-3’ |
| mTTLL13 | 5’- GGCCTGAAGGAAGTAGGGGA-3’ | 5’- CATGCCAGGGAAGTGGTTGA-3’ |
| mActb | 5’- TGACGGGGTCACCCACACTGTGCCCATCTA-3’ | 5’- CTAGAAGCATTTGCGGTGGACGATGGAGGG-3’ |
| mRex1 | 5’- AAGGGGACGAAGCAAGAGAAG-3’ | 5’- TCTGGGTTGTACGGGTCATAG-3’ |
| mEsrrb | 5’-GCACCTGGGCTCTAGTTGC-3’ | 5’-TACAGTCCTCGTAGCTCTTGC-3’ |
| mSox2 | 5’-GCGGAGTGGAAACTTTTGTCC-3’ | 5’-GCCGCCTGCAAGTAATGAG-3’ |
| mLin28A | 5’-GGCATCTGTAAGTGGTTCAACG-3’ | 5’-CCCTCCTTGAGGCTTCGGA-3’ |
| mNanog | 5’-TCTTCCTGGTCCCCACAGTTT-3’ | 5’-GCAAGAATAGTTCTCGGGATGAA-3’ |
| hREX1 | 5’- TAGAATGCGTCATAAGGGGTGA- 3’ | 5’- CTGCAGTGTGGGTTTCGGGCA-3’ |
| hSOX2 | 5’-GCCGAGTGGAAACTTTTGTCG - 3’ | 5’- TCTTGCCTGTCATGTACTCAGAA-3’ |
| hLIN28A | 5’- AGCGCAGATCAAAAGGAGACA- 3’ | 5’-CCTCTCGAAAGTAGGTTGGCT -3’ |
| hESRRB | 5’-ATCAAGTGCGAGTACATGCTC - 3’ | 5’- CGCCTCCGTTTGGTGATCTC-3’ |
| hNANOG | 5’-CAAAGGCAAACAACCCACTT - 3’ | 5’-TCTGCTGGAGGCTGAGGTAT -3’ |
| hACTB | 5’- CATGTACGTTGCTATCCAGGC- 3’ | 5’-CTCCTTAATGTCACGCACGAT -3’ |
| mRex1 pro  mEsrrb pro  mSox2 pro  mLin28A pro  mNanog pro | 5’-GAGGTACTGAGATGTGACTGAGTCTCA-3’  5’- CCAACCAGAAGTGGGTCTTGTTCCT-3’  5’- TTTTCGTTTTTAGGGTAAGGTACTGGGAAG-3’  5’-ACGTTAGGCGAGTTGAGC-3’  5’- ATCCACCTGCCTCTGCCGCCTAA-3’ | 5-CTCCTTGGACCCCTCCCTTTTTAGATG-3′  5’-TGTGGAAGGATCCTGGACACAGAT-3’  5’-CCACGTGAATAATCCTATATGCATCACAAT-3’  5’-ATATAAGCACTGCGATCATCC-3’  5’- GCATTGGTGTTTTGCCTGCATGG-3’ |
